# Pharyngeal Cervical Neurinoma: dysphonia and dysphagia

**DOI:** 10.1016/S1808-8694(15)30136-1

**Published:** 2015-10-19

**Authors:** Carla Maffei, Maria Ines Rebelo Gonçalves, Marçal Motta de Mello, Jaerilson h. kluppel, Paulo Antonio Monteiro Camargo

**Affiliations:** a M.S. in Communications Disorders - Tuiuti University - Paraná - PhD student in Stomatology - Pontifícia Universidade Católica do Paraná. Volunteer Researcher - M.S. Program of Communications Disorders - Tuiuti University - Paraná; b Post-Doctorate degree - California University, Adjunct Professor of the M.S. Program in Communications Disorders - Tuiuti University - Paraná; c Specialist in Oral Motricity/Dysphagia - Tuiuti University - Paraná. Physician in Charge of the swallowing videofluoroscopy exams sector - Red Cross Hospital - Paraná; d DDS. Specialist in Periodontology - University of Araçatuba, São Paulo; e PhD, Professor/Director -Centro Avançado de ORL/Cérvico-facial. Universidade Tuiuti do Paraná Centro Avançado de Otorrinolaringologia - CAO Hospital da Cruz Vermelha do Paraná Hospital São Vicente do Paraná

**Keywords:** dysphonia, dysphagia, electromyography, neurinoma

## INTRODUCTION

More advanced neuromas originate from the cranial and spinal nerve roots, and they manifest symptoms as slow and progressive spine compression, and partial or total surgical removal is indicated^1^. Cranial pairs V, VII, IX, X, XI and XII act directly on speech production and swallowing^2^, ^3^ and, when involved, may interfere in the patient’s life quality, especially the social, nutritional and pulmonary aspects. The more these nerves are involved, the higher is the risk of developing aspiration pneumonia^4^, and the multidisciplinary care is fundamental in order to treat these patients and minimize resection sequelae^5^, ^6^.

Our goal was to check the results of speech, mastication and swallowing rehabilitation in a case of neck and pharyngeal neuroma by means of exams: laryngeal, swallowing video fluoroscopy, and electromyography of masticatory muscles.

## CASE PRESENTATION

A 38 year old patient, that after suffering a resection of a neck and pharyngeal neuroma that involved the neck region and the carotid artery, complained of dysphonia, dysphagia and chewing difficulties.

In our evaluation we noticed tongue atrophy and reduction of labial mobility on the right side, associated with reduced tongue elevation. The videolaryngoscopy showed paralysis of the hemilarynx and also of the right vocal fold in total abduction, reduction in soft palate muscle contraction on the right side and a blowy voice pattern. After a type I thyroplasty, she presented with a posterior midline triangular vocal fold slit with partial vocal improvement.

During swallowing videofluoroscopy we noticed for all types of food material: premature food escape; food residues in the oral cavity; difficulty in transporting the food from the oral phase to the pharynx and food stasis in the piriform fossae and piriform recesses.

Electromyography showed a reduction in temporal and masseter muscle activity on the right side.

Speech therapy rehabilitation happened in 24 sessions, with isotonic and isometric myofunctional exercises of the tongue and masticatory muscles (masseter and temporalis) and the utterance of vowel /i/ sustained with hands in hook-shape associated with neck flexion to the right.

## DISCUSSION

Table 1 depicts the pre and post rehabilitation findings. After speech therapy, the new tests showed: improvements in tongue strength, tonus and mobility; no premature food escape; increase in pharynx constriction muscles contraction, with residue reduction in the piriform fossae; increase in right side temporal and masseter muscles activity and unaltered voice quality.

**Table 1:** Otorhinolaryngological and Speech Therapy findings in the oral cavity and larynx, videofluoroscopy of the oral and pharyngeal phases of swallowing and electromyographic findings of the masticatory muscles before and after speech therapy.

Pre and post speech therapy findings		
Right side larynx paralysis	Present	Present
Mid-Posterior triangular glottal slit	Present	Present
Right side soft palate mobility reduction	Present	Present
Right side tongue atrophy	Present	Absent
Tongue elevation	Absent	Present
Tongue projection deviation	Present	Absent
Tongue strength in counter resistance	Absent	Present
Tongue widening and thinning	Absent	Present
Residue in the oral cavity	Present	Absent
Motor oral difficulty	Present	Absent
Premature escape	Present	Absent
Number of swallowing efforts for 5ml of liquid	3	1
Number of swallowing efforts for 10ml of liquid	3	2
Number of swallowing efforts for 5ml of paste	4	2
Number of swallowing efforts for 10ml of paste	5	3
Number of swallowing efforts for solid food	5	2
Stasis in piriform fossae	Moderate	Mild
Stasis in piriform recess	Moderate	Mild
Right side temporal muscles activity	Reduced	Increased
Right side masseter muscles activity	Reduced	Increased

It is important to highlight that the exercises selected for the speech therapy rehabilitation in this patient were those that, besides indicated, presented the best results during the therapeutic tests.

The vocal exercise carried out was then selected because it was the only one that caused a mild reduction in the glottal slit, and also a mild increase in voice blowing pattern during its performance. Along speech therapy, other vocal exercises were selected, associated or not to neck postural maneuvers, but also without good results as far as vocal quality is concerned.

## FINAL REMARKS

There was a significant improvement in mastication, swallowing, tongue muscle activity and masticatory muscles after speech therapy rehabilitation, although voice quality remained unaltered.
